# Comparison of soft tissue balancing, femoral component rotation, and joint line change between the gap balancing and measured resection techniques in primary total knee arthroplasty: A meta-analysis

**DOI:** 10.1097/MD.0000000000005006

**Published:** 2016-09-30

**Authors:** Young-Wan Moon, Hyun-Jung Kim, Hyeong-Sik Ahn, Chan-Deok Park, Dae-Hee Lee

**Affiliations:** a Department of Orthopaedic Surgery, Samsung Medical Center, Sungkyunkwan University School of Medicine; b Department of Preventive medicine, Korea University, College of Medicine, Seoul, Korea.

**Keywords:** gap balancing, measured resection, total knee arthroplasty

## Abstract

**Background:**

This meta-analysis was designed to compare the accuracy of soft tissue balancing and femoral component rotation as well as change in joint line positions, between the measured resection and gap balancing techniques in primary total knee arthroplasty.

**Methods:**

Studies were included in the meta-analysis if they compared soft tissue balancing and/or radiologic outcomes in patients who underwent total knee arthroplasty with the gap balancing and measured resection techniques. Comparisons included differences in flexion/extension, medial/lateral flexion, and medial/lateral extension gaps (LEGs), femoral component rotation, and change in joint line positions. Finally, 8 studies identified via electronic (MEDLINE, EMBASE, and the Cochrane Library) and manual searches were included. All 8 studies showed a low risk of selection bias and provided detailed demographic data. There was some inherent heterogeneity due to uncontrolled bias, because all included studies were observational comparison studies.

**Results:**

The pooled mean difference in gap differences between the gap balancing and measured resection techniques did not differ significantly (−0.09 mm, 95% confidence interval [CI]: −0.40 to +0.21 mm; *P* = 0.55), except that the medial/LEG difference was 0.58 mm greater for measured resection than gap balancing (95% CI: −1.01 to −0.15 mm; *P* = 0.008). Conversely, the pooled mean difference in femoral component external rotation (0.77°, 95% CI: 0.18° to 1.35°; *P* = 0.01) and joint line change (1.17 mm, 95% CI: 0.82 to 1.52 mm; *P* < 0.001) were significantly greater for the gap balancing than the measured resection technique.

**Conclusion:**

The gap balancing and measured resection techniques showed similar soft tissue balancing, except for medial/LEG difference. However, the femoral component was more externally rotated and the joint line was more elevated with gap balancing than measured resection. These differences were minimal (around 1 mm or 1°) and therefore may have little effect on the biomechanics of the knee joint. This suggests that the gap balancing and measured resection techniques are not mutually exclusive.

## Abstract 2

Supplemental Digital Content is available in the text

## 1. Introduction

Primary total knee arthroplasty (TKA) is an established surgical method for treating end-stage knee osteoarthritis and rheumatoid arthritis. The growing number of elderly individuals worldwide has meant that, in the United States alone, approximately one million primary TKAs are performed per year.^[1]^ Although primary TKA is the most popular surgical treatment of end-stage knee arthritis, the 15-year survival rate is 90% to 95% and 10% to 20% of patients are not satisfied with the results,^[2–4]^ suggesting the difficulties associated with primary TKA and the importance of good surgical technique. There are several prerequisites for the success of primary TKA, including neutral alignment and good soft tissue balancing, correct rotation of the femoral component, and minimal change in joint line after surgery.^[5–7]^ Equalized rectangular joint gaps after osteotomy at 90° flexion and full extension are indicators of good soft tissue balancing and are dependent on accurate bone resection combined with stepwise release of tight soft tissue.^[8–10]^ Femoral component rotation is also associated with good soft tissue balancing, especially at 90° flexion, with malrotation associated with patellofemoral and tibiofemoral instabilities, knee pain, arthrofibrosis, and abnormal kinematics.^[11–13]^ Restoration of the preoperative joint line also correlates with good soft tissue balancing; an extremely elevated joint line after TKA can have adverse effects on collateral ligament function and the patellofemoral joint mechanism.^[14]^

The standard surgical techniques for TKA utilize 2 distinct methods of prosthesis implantation: measured resection and gap balancing. Theoretically, measured resection and all bone resections are performed first, including cutting of the femur and tibia, and then soft tissue balancing is performed. However, in the gap balancing technique, soft tissue balancing is performed before femoral bone cutting.^[15,16]^The differences in these approaches may affect femoral component rotation and change in joint line position.^[15,17]^ Few studies, however, have directly compared outcomes following measured resection and gap balancing. Thus, the optimal method for achieving appropriate soft tissue balancing and femoral component rotation, with minimal joint line position change, remains unclear. This meta-analysis was therefore designed to compare outcomes in patients who underwent TKA using the measured resection and gap balancing techniques. Outcomes analyzed included the accuracy of soft tissue balancing and femoral component rotation as well as change in joint line position. It was hypothesized that the 2 approaches would result in similar soft tissue balancing, femoral component rotation, and joint line position change.

## 2. Materials and methods

This meta-analysis was performed according to the guidelines of the preferred reporting items for systematic reviews and meta-analysis statement. There were no ethical approval and patient written informed consent because this study was a meta-analysis based on the published studies.

### 2.1. Data and literature sources

This study was based on Cochrane Review Methods. Multiple comprehensive databases, including MEDLINE (January 1, 1976 to September 30, 2015), EMBASE (January 1, 1985 to September 30, 2015), and the Cochrane Library (January 1, 1987 to September 30, 2015), were searched for studies that compared soft tissue balancing, femoral component rotation, and joint line change, in patients who underwent TKA using gap balancing and measured resection techniques. There were no restrictions on language or year of publication. Search terms used in the title, abstract, and keywords fields included (“arthroplasty, replacement, knee” [Mesh] OR “total knee arthroplasty” [tiab]) AND “gap balancing” [tiab] OR “measured resection” [tiab]). There were no restrictions on language or year of publication. After the initial electronic search, additional relevant articles and bibliographies from identified studies were hand searched. Articles identified were assessed individually for inclusion.

### 2.2. Study selection

Study inclusion was decided independently by 2 reviewers, based on predefined selection criteria. Titles and abstracts were read; if suitability could not be determined, the full article was evaluated. The gap balancing technique was usually performed by first cutting the tibia and then the femur according to the degree of soft tissue balancing required to achieve rectangular flexion and extension gaps. In the measured resection technique, by contrast, all bone cutting was performed first on both the femur and tibia, after which soft tissue balancing was performed to achieve rectangular extension and flexion gaps by soft tissue release. In other words, in the gap balancing technique soft tissue balancing was performed before femoral cutting, whereas in the measured resection technique soft tissue balancing was performed after all bone cutting, including that of the femur, was completed.

Studies were included in the meta-analysis if they compared soft tissue balancing or radiologic outcomes in patients who underwent TKA with gap balancing and measured resection techniques; and they directly compared at least 1 parameter related to surgical outcomes, including gap differences (eg, flexion/extension, medial/lateral flexion, or medial/LEG), femoral component rotation on postoperative computed tomography, and change in joint line before and after surgery. After the completion of bone cutting, the extension and flexion gaps were measured on the medial and lateral sides at full extension and at 90° flexion, using a spreading and measuring device (similar to a lamina spreader) with a torque meter, tensor, and sliding rule. These 4 gaps were defined as the medial extension gap, medial flexion gap (MFG), LEG, and lateral flexion gap (LFG). The knees with all 4 gap differences of 3 mm or less were defined as having rectangular, well-balanced gaps that were considered acceptable for soft tissue balancing. Femoral component rotation was defined as the angle subtended between the surgical transepicondylar axis and the prosthetic posterior condylar tangential line of the femoral component. The joint line was defined as the perpendicular distance from the tibial tubercle or tip of the fibular head to the distal femur (preoperatively) or the tangential line of the femoral component (postoperative), with the change in joint line calculated as the distance between these 2 lines. Studies were also included if they reported the number of patients in the gap balancing and measured resection groups, and the means and standard deviations in each group of gap differences, femoral component rotation, and change in joint line, and used adequate statistical methods to compare these parameters.

### 2.3. Data extraction

Two reviewers independently recorded data from each study using a predefined data extraction form, which is shown in a supplementary file. Any disagreement unresolved by discussion was reviewed by a third investigator. Variables recorded included means and standard deviations of gap differences, femoral component rotation, and change in joint line from before to after surgery; and the sample size of each group. If these variables were not mentioned in the articles, the authors of the study were contacted by email to request the data.

### 2.4. Assessment of methodological quality

As recommended by the Cochrane Non-Randomized Studies Methods Working Group, the methodological quality of each study was evaluated by 2 independent investigators using the Newcastle-Ottawa Scale, adjusted to a scale that included only low (1 star), high, and unclear bias. Each study was judged on 3 criteria, which are as follows: the selection of the study groups, the comparability of the groups, and the ascertainment of either the exposure or the outcome of interest for case–control and cohort studies. Any unresolved disagreements between reviewers were resolved by consensus or by consultation with a third investigator. Publication bias was not assessable in these trials, because test for funnel plot asymmetry are generally performed only when at least 10 studies are included in the meta-analysis. As our analysis included only 8 studies, tests for asymmetry were not performed as they would be unable to differentiate chance from asymmetry.

### 2.5. Statistical analysis

The main outcomes of the meta-analysis were the mean differences in gap differences, femoral component rotation, and change of joint line from before to after surgery in groups of patients who underwent TKA using gap balancing and measured resection techniques. Mean differences and 95% CIs were calculated for continuous outcomes. In terms of gap differences, 3-subgroup analyses were performed for mean differences of flexion/extension gaps, medial/LFGs, and medial/LEGs. These values were analyzed with a random effects model. Interrater reliability in assessing methodological quality was evaluated by kappa (к), with values of 0.40 or less, 0.41–0.60, 0.61–0.80, and 0.81–1.00 indicating no, moderate, substantial, and almost perfect agreement, respectively. Heterogeneity was determined by estimating the proportion of between-study inconsistencies due to actual differences between studies, rather than differences due to random error or chance, using the *I*^2^ statistic, with values of 25%, 50%, and 75% indicating low, moderate, and high heterogeneity, respectively. All statistical analyses were performed using RevMan version 5.2 (Cochrane Collaboration, UK) and Stata/MP 14.0 (Stata Corp, College Station, TX).

## 3. Results

### 3.1. Identification of studies

**Figure 1:**
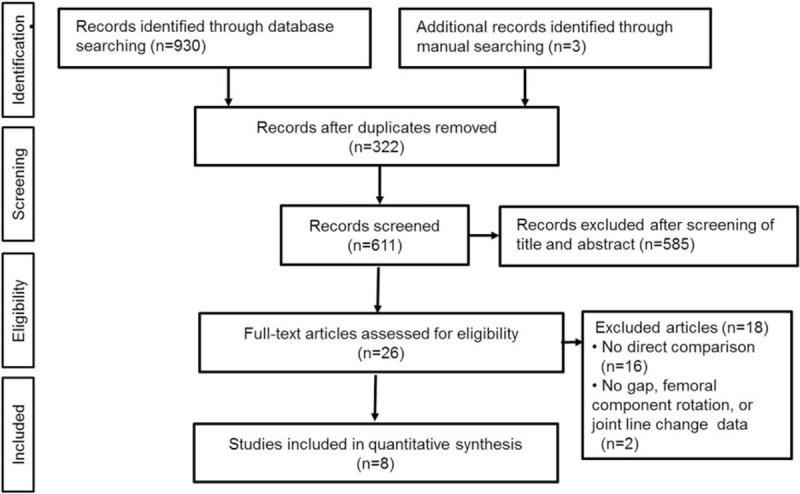
PRISMA flow diagram of the identification and selection of the studies included in this meta-analysis. PRISMA = preferred reporting items for systematic reviews and meta-analyses.

Figure 1 shows the details of study identification, inclusion, and exclusion. Electronic searches of PubMed (MEDLINE), EMBASE, and the Cochrane Library yielded 465, 419, and 46 studies, respectively. Three additional publications were identified through manual searching. After removing 322 duplications, 611 studies remained; of these, 585 were excluded based on reading of the abstracts and full-text articles. An additional 18 studies were excluded based on unusable information and inappropriate group comparisons. After applying these criteria, 8 studies were finally included in this meta-analysis.

### 3.2. Study characteristics and quality appraisal

Of the 8 included studies, one compared all end-point parameters, including gap symmetry, femoral component rotation, and joint line change, between the gap balancing and measured resection groups; 1 compared gap symmetry and joint line change; and 6 studies each compared 1 parameter among the 3 categories; that is, gap symmetry, femoral component rotation, or joint line change (Table 1).

**Table 1:**
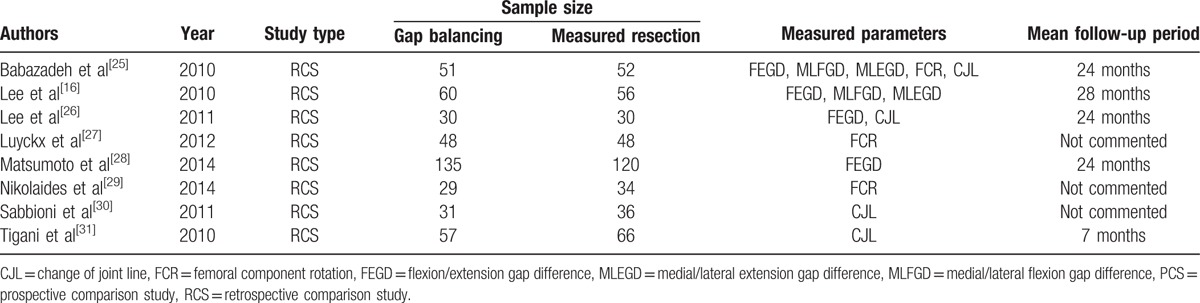
Study characteristics.

All 8 studies included in this meta-analysis showed a low risk of selection bias and provided detailed demographic data. None assessed possible confounding factors, and none mentioned the percentage of patients evaluated, relative to all patients who underwent TKA at that institution. All studies included in this meta-analysis were deemed as having a high risk of bias as determined by adequacy of follow-up (Table 2). Interrater reliabilities (к values) for all items of newcastle-ottawa scale was ranged from 0.72 to 0.87, indicating at least more than substantial agreement between 2 investigators.

**Table 2:**
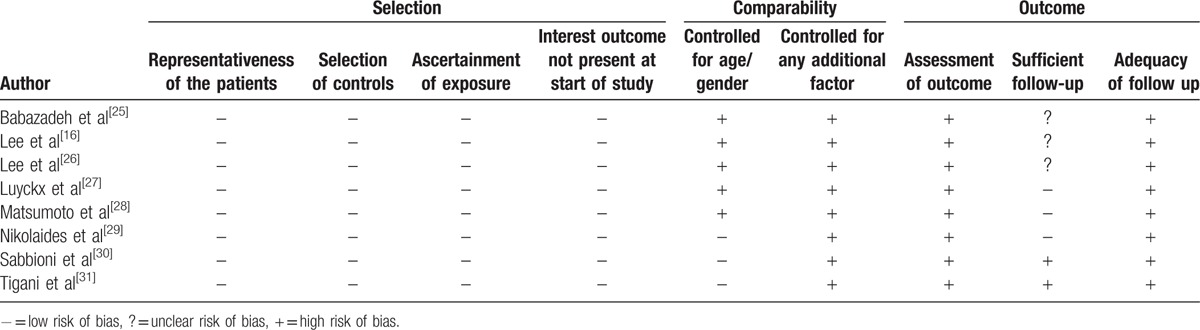
Risk of bias summary: review authors’ judgments about the risk of bias for items in each included study.

### 3.3. Gap symmetry

**Figure 2:**
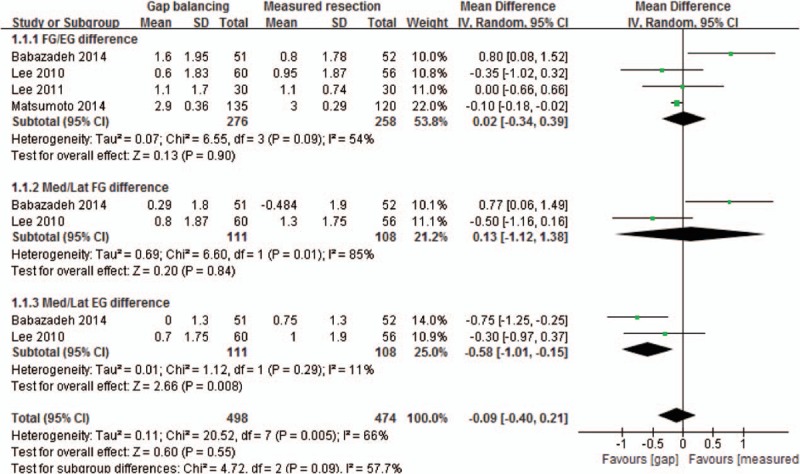
Forest plot showing mean differences in gap symmetries, including the flexion/extension gap difference, medial and lateral flexion gap difference, and medial and lateral extension gap difference, between the gap balancing and measured resection techniques.

Of the 8 studies, 4 compared flexion/extension gap differences in 276 knees that underwent gap balancing and 258 that underwent measured resection techniques. Of these 4 studies, 2 also compared differences in the medial and lateral flexion and extension gaps between patients who underwent TKA using the 2 techniques. The pooled mean difference in gap differences between the gap balancing and measured resection techniques was −0.09 mm (95% computed tomography [CI]: −0.40 to +0.21 mm; *P* = 0.55; *I*^2^ = 66%), a difference that was not statistically significant. Subgroup analysis showed a lack of significance in mean differences in flexion/extension (0.02 mm, 95% CI: −0.34 to +0.39 mm; *P* = 0.90; *I*^2^ = 54%) and medial/lateral flexion (0.13 mm, 95% CI: −1.12 to +1.38 mm; *P* = 0.84; *I*^2^ = 85%) gap differences. In contrast, the mean difference in medial/LEG difference was significantly greater in patients undergoing the measured resection than the gap balancing technique (0.58 mm, 95% CI: −1.01 to −0.15 mm; *P* = 0.008; *I*^2^ = 11%, Fig. 2).

### 3.4. Femoral component rotation

**Figure 3:**
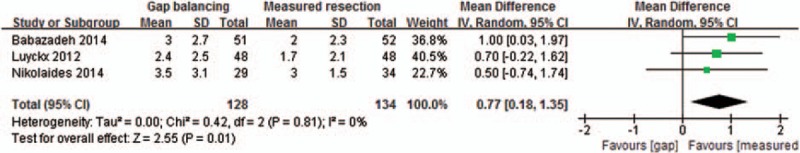
Forest plot showing mean differences in femoral component rotation between the gap balancing and measured resection techniques.

Three of the 8 included studies compared femoral component rotation on postoperative computed tomography in 128 knees that underwent gap balancing and 134 that underwent measured resection. The pooled mean difference in femoral component rotation between the 2 techniques was 0.77° (95% CI: 0.18° to 1.35°; *P* = 0.01; *I*^2^ = 0%, Fig. 3), indicating that the femoral component showed significantly greater external rotation in patients who underwent TKA using the gap balancing than the measured resection technique.

### 3.5. Change of joint line

**Figure 4:**
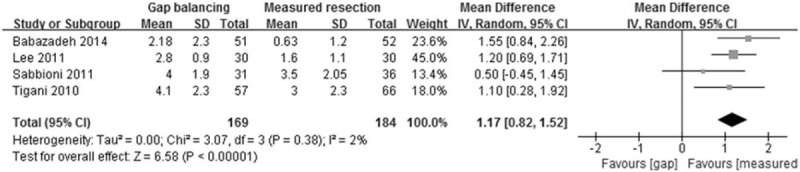
Forest plot showing mean differences in elevation of joint line after TKA using the gap balancing and measured resection techniques. TKA = total knee arthroplasty.

Four of the 8 studies compared changes in joint line in 169 knees that underwent gap balancing and 184 that underwent measured resection. The pooled mean difference in femoral component rotation between these 2 groups was 1.17 mm (95% CI: 0.82 to 1.52 mm; *P* < 0.001; *I*^2^ = 2%, Fig. 4), indicating that joint line elevation after TKA was 1.17 mm greater in patients who underwent gap balancing than measured resection.

## 4. Discussion

The most important findings of this meta-analysis were that the gap balancing and measured resection techniques yielded similar gap symmetries, except for the difference between medial and LEGs. However, the gap balancing technique resulted in greater external rotation and joint line change than the measured resection technique.

In patients undergoing TKA, soft tissue was balanced through the step-by-step release of soft tissue of the tight portion around the knee. Soft tissue release during TKA affects both medial and lateral flexion and extension gaps simultaneously,^[18]^ although the magnitude of such gap changes is not always equal. A study investigating the effects of the specific release of medial soft tissue on joint gap change in varus osteoarthritic knees reported that all steps of soft tissue release for specific anatomical structures, including the medial or posteromedial joint capsule, the semimembranosus tendon, and the tibial insertion of the medial collateral ligament, increased both the flexion and extension gaps on both the medial and lateral sides an average of 0.3 to 3.8 mm.^[19]^ That study also showed that the standard deviation in joint gap change was greater for flexion than extension gaps, especially on the medial side, indicating that a more varus tendency resulted in a greater increase in flexion than extension gap, particularly in the medial compartment. These varied and unpredictable changes in MFG indicate that the differences in medial and LFGs did not differ significantly in patients undergoing TKA using gap balancing and measured resection techniques. Because of the natural laxity of the lateral compartment, a lateral gap slightly larger than a medial gap is close to physiologic conditions.^[20]^ Therefore, the finding in the current meta-analysis, that the minimal difference in extension gap between the medial and lateral sides differed significantly between patients who underwent gap balancing and measured resection techniques, was due to the gap balancing technique ascribing more importance to gap symmetry than does the measured resection technique. In contrast, medial and LFG differences were similar with the 2 techniques. This finding may be caused by the vulnerability to medial soft tissue release of medial joint gap change at 90° flexion, which may result in the over-release of medial soft tissue, resulting in a greater increase in the medial than the LFG. However, the results of the current meta-analysis showed similar gap symmetry, except for the medial/LEG, suggesting that both the gap balancing and measured resection techniques aimed to achieve symmetric joint gaps and that both techniques may therefore not be mutually exclusive.^[21]^

The gap balancing technique has a theoretical disadvantage, in that it does not consider the natural laxity of the lateral compartment of the knee joint. The lateral compartments are normally slacker than the medial compartments, especially during flexion.^[22]^ Therefore, if tensors are used to rotate the femur until the medial and lateral collateral ligaments are equally loaded, the lateral joint space may be wider than the medial joint space. This situation would force the tibia into a more varus position, resulting in the femur being in internal rotation relative to the tibia.^[23]^ The femoral component must therefore be rotated more externally, so that the wide LFG is equal to the relatively narrow MFG. This may explain our result showing that external rotation was greater with the gap balancing than the measured resection technique. However, the amount of external rotation with the gap balancing technique was less than 1°, suggesting that the difference between the 2 methods was not clinically important.

Three studies included in this meta-analysis that compared femoral component rotation between the gap balancing and measured resection techniques did not find a significant difference between these 2 methods. In these 3 studies, gap balancing showed a 0.5° to 1.0° greater external rotation than the measured resection technique. This may be a type II (false negative) error due to a lack of adequate power resulting from the small sample size in each study.^[24]^ However, by pooling data from these 3 studies, we found a statistically significant difference in femoral component rotation between the 2 techniques. Sufficient statistical power resulted from pooling the data of individual studies, minimizing type II errors.

The greater elevation of the joint line observed with gap balancing may have been due to this method showing greater prioritization of gap symmetry than the measured resection technique. A recent study^[25]^ compared the thickness of the distal femur and tibia after cutting, tibial polyethylene liner size, and joint line change in patients undergoing TKA using these 2 methods. That study demonstrated that gap balancing significantly increased the joint line due to greater cutting of the distal femur and tibia as well as a thicker polyethylene liner. The greater joint line elevation of the gap balancing technique would therefore be offset by its more symmetric gap balance.

This study had several limitations. All of the studies included in this meta-analysis were observational comparison studies. Therefore, there was some inherent heterogeneity due to uncontrolled bias. In addition, studies differed in surgical approaches and the use of prostheses. Specifically, when measuring the joint gap, the applied tension differed among studies. These factors may explain, at least in part, some of the heterogeneities in the results of this meta-analysis. However, because all studies used the same methods to measure femoral component rotation and joint line change, heterogeneity was likely minimized.

Despite these limitations, the current meta-analysis found that the gap balancing and measured resection techniques yielded similar soft tissue balancing, except for a minimal extension gap difference (0.58 mm) of the medial and lateral compartments, and a trend toward a slightly greater external rotation of the femoral component (0.77°) and joint line elevation (1.17 mm) in the gap balancing compared to the measured resection technique. These differences were minimal (around 1 mm or 1°) and therefore may have little effect on knee biomechanics, suggesting that the gap balancing and measured resection techniques are not mutually exclusive. However, future long-term prospective studies are warranted to more firmly establish the effect of these differences on clinical outcomes, because TKA is a detailed procedure with a narrow forgiveness range.

## 5. Conclusions

In conclusion, the gap balancing and measured resection techniques showed similar soft tissue balancing except for the extension gap difference of the medial and lateral compartments. However, the femoral component was more externally rotated and the joint line was more elevated in patients who underwent primary TKA using the gap balancing than the measured resection technique.

## Supplementary Material

Supplemental Digital Content
