# Supplementary material for: Comparison of soft tissue balancing, femoral component rotation, and joint line change between the gap balancing and measured resection techniques in primary total knee arthroplasty: A meta-analysis
Source: Medicine (Baltimore). 2016 Sep 30;95(39):e5006. doi: 10.1097/MD.0000000000005006 (PMC5265955; doi:10.1097/MD.0000000000005006)
Supplement: Supplemental Digital Content [file medi-95-e5006-s001.doc]

# Data extraction form

|  | | **Description as stated in report/paper** | **Location in text** |
| --- | --- | --- | --- |
| 1. Title of paper | |  |  |
| 1. Publication type   ***(e.g. full report, abstract, letter)*** | |  |  |
| 1. Types of outcome measures | |  |  |
| 1. Total sample no. in study | |  |  |
| 1. Method/s of recruitment of participants | |  |  |
| 1. No.of each group | |  |  |
| 1. Gap differences   **Or 4 Gaps** | |  |  |
| 1. Femoral component rotation | | ISI/BPI/CD index |  |
| 1. Change in joint line | |  |  |
| 1. Key conclusions of study authors | |  |  |
| 1. Notes: |  | | |
